# The CAnadian Network for Psychedelic-Assisted Cancer Therapy (CAN-PACT): A Multi-Phase Program Overview

**DOI:** 10.3390/curroncol33010007

**Published:** 2025-12-22

**Authors:** Linda E. Carlson, Harriet Richardson, Ron Shore, Christopher P. Albertyn, Lynda G. Balneaves, Alan Bates, Margot Burnell, Harvey Max Chochinov, David Clements, Julie Deleemans, Hilary Horlock, Jean Mathews, Michael McKenzie, Chantal Savard, Claudio N. Soares, Wei Tu, Monnica Williams

**Affiliations:** 1 Division of Psychosocial Oncology, Department of Oncology, Cumming School of Medicine, University of Calgary, Calgary, AB T2N 2T8, Canadachantal.savard1@ucalgary.ca (C.S.); 2 Sinclair Cancer Research Institute, Divisions of Canadian Cancer Trials Group & Cancer Care and Epidemiology, Queen’s University, 10 Stuart Street, Kingston, ON K7L 3N6, Canada; hrichardson@ctg.queensu.ca; 3 Centre for Psychedelics Health and Research at Providence Care, Kingston, ON K7L 4X3, Canada; shore.r@queensu.ca (R.S.); claudio.soares@kingstonhsc.ca (C.N.S.); 4 Department of Psychiatry, Queen’s Health Sciences, Queen’s University, Kingston, ON K7L 5G2, Canada; 5 College of Nursing, Rady Faculty of Health Sciences, University of Manitoba, Winnipeg, MB R3T 2N2, Canada; lynda.balneaves@umanitoba.ca; 6 Supportive Care, BC Cancer Agency, Vancouver, BC V5Z 4E6, Canada; alan.bates@bccancer.bc.ca; 7 Department of Psychiatry, University of British Columbia, Vancouver, BC V6T 0A6, Canada; 8 Saint John Regional Hospital, Saint John, NB E2L 4L2, Canada; 9 Department of Psychiatry, Max Rady College of Medicine, University of Manitoba, 750 Bannatyne Ave, Winnipeg, MB R3E 0T4, Canada; 10 CancerCare Manitoba Research Institute, Winnipeg, MB R3E 0V9, Canada; 11 Department of Health Sciences, Carleton University, Ottawa, ON K1S 5B6, Canada; 12 Patient Partner with Lived Experience, Vancouver, BC, Canada; 13 Division of Palliative Medicine, Departments of Medicine and Oncology, Queen’s University, Kingston, ON K7L 3N6, Canada; jean.mathews@kingstonhsc.ca; 14 Radiation Therapy Program, BC Cancer, Vancouver, BC V5Z 1G1, Canada; mmckenzi@bccancer.bc.ca; 15 Canadian Cancer Trials Group, Queen’s University, Kingston, ON K7L 3N6, Canada; 16 School of Psychology, University of Ottawa, Ottawa, ON K1N 6N5, Canada

**Keywords:** cancer, psychedelics, psilocybin, mindfulness, demoralization, fear of death and dying, clinical trials, patient-oriented research

## Abstract

The CAnadian Network for Psychedelic-Assisted Cancer Therapy (CAN-PACT) was launched in 2025 to address urgent gaps in supportive care for Canadians with cancer experiencing demoralization syndrome (loss of meaning, dysphoria, disheartenment, helplessness, a sense of failure) and related psychosocial distress. CAN-PACT has six major objectives: (1) to develop a national interdisciplinary research and practice network; (2) to set research priorities through structured stakeholder engagement; (3) to develop and provide PAT training and education for clinicians, researchers, and patients; (4) to pilot test the feasibility of intervention and assessment procedures; (5) to conduct a multi-center, randomized controlled trial of PAT for people with advanced cancer; and (6) to inform and influence healthcare policy on PAT in Canada. We discuss the background and need for PAT in cancer, describe challenges currently limiting its use, and outline CAN-PACT’s strategy for building capacity, generating Canadian evidence, and preparing the oncology healthcare environment for potential implementation. This manuscript presents a summary overview of CAN-PACT as a multi-objective research program; detailed protocols for each discrete study component will be published separately as the research program progresses. Through environmental scans, national engagement, targeted training, rigorous research, and ongoing collaboration with policymakers, CAN-PACT aims to enable equitable access to safe, evidence-based PAT for people with advanced cancer in Canada’s publicly funded cancer centers.

## Simple Summary

People living with cancer often struggle with distressing feelings such as loss of hope, anxiety, and a sense of meaninglessness and demoralization that negatively impacts quality of life. While there are psychosocial interventions for these symptoms, challenges such as access, delayed onset of treatment effects, and incomplete responses highlight the need for new, fast-acting, potentially synergistic therapies to better manage these distressing symptoms. Research suggests that psychedelic-assisted therapies, often using psilocybin in combination with psychological support, may offer accelerated and lasting relief for many people. The CAN-PACT project brings together a Canadian network of researchers, clinicians, policymakers, and people affected by cancer to investigate the safety, effectiveness, and real-world integration of psychedelic-assisted therapy (PAT) in cancer care. By emphasizing diversity, person-centered approaches, and training for healthcare providers, this innovative work aims to lay the foundation for future studies and inform policies that could expand supportive treatment options for people with cancer experiencing demoralization.

## 1. Introduction

Cancer remains the leading cause of death in Canada, with a projected 247,100 new cancer cases and 88,100 cancer deaths in 2024 alone, an increase from 2023 [1]. Approximately 30% to 40% of incident cancer cases in Canada are stage III or IV—a weighted average derived from the most frequently diagnosed cancers in Canada, including the high proportion of late-stage diagnoses in lung and colorectal cancers and the lower rates of late-stage diagnoses in breast and prostate cancers. While people living with cancer at any stage often suffer high psychosocial burden [2,3], the cohort of people with advanced cancer often experience higher rates of physical, emotional, and psychological distress, including significant existential anxiety, depression, and demoralization. Conventional psychopharmacological treatments for depression and anxiety, such as anti-depressants, often have limited efficacy for existential distress and demoralization, leaving patients with unmet needs in palliative care. Psychedelic-Assisted Therapy (PAT) is a promising addition to psychosocial oncology care, potentially alleviating suffering and improving quality of life for patients with advanced cancer. However, significant challenges prevent access to PAT, including a lack of capacity for both research and clinical services, limited safety and efficacy data, and regulatory restrictions on psychedelics such as psilocybin. The goal of the CAN-PACT initiative is to address these barriers by building the infrastructure, capacity, and research foundation necessary to implement evidence-based PAT for people living with cancer across Canada.

In this manuscript, we outline the structure and rationale of CAN-PACT as an integrated, multi-phase research program (rather than presenting a full protocol for any single clinical trial). The six interdependent CAN-PACT objectives unfold sequentially and iteratively, with earlier phases of network development, priority setting, training, and feasibility testing intentionally informing the design of the definitive clinical trial. Formal, detailed methodological protocols for each discrete study component—including the priority setting partnership, pilot feasibility study, and multi-site randomized controlled trial (RCT)—will be published separately as their respective phases progress. This approach reflects best practices in complex health systems intervention research, where preliminary investigation necessarily precedes large-scale efficacy trials.

### 1.1. Psychosocial Experience of Advanced Cancer

People with advanced cancers often suffer disproportionately from the psychosocial burden of their illness, with many experiencing symptoms of depression, existential anxiety, fear of death, and demoralization syndrome (DS) [4]. DS represents a clinically significant state of existential distress that extends beyond simple sadness or discouragement, characterized by a profound sense that life has lost its meaning and purpose in the face of overwhelming circumstances. Critically, DS differs from depression in important ways. While depression is characterized by pervasive anhedonia, neurovegetative symptoms, and nihilistic thinking, demoralization is primarily an existential crisis marked by hopelessness about one’s circumstances rather than oneself [5]. Patients experiencing demoralization may retain the capacity to experience pleasure in the moment but lose the ability to anticipate future pleasures, whereas depressed patients lose both. A significant proportion of cancer patients experiencing high demoralization do not meet criteria for major depression, yet they require equally urgent clinical attention. Kissane et al. developed a tool—the ‘Demoralization Scale’ [6]—identifying five factors that characterize demoralization syndrome: loss of meaning, dysphoria, disheartenment, helplessness, and a sense of failure. Approximately 13–23% of people with advanced cancers experience DS [7], which remains difficult to treat and exacerbates suffering, compounding physical symptoms and complicating palliative care. The adverse consequences of untreated demoralization in cancer patients are substantial and multifaceted. Most critically, demoralization is an independent predictor of suicidal ideation, with strong evidence demonstrating a bi-directional relationship whereby suicidal ideation is both an associated factor and an outcome of demoralization. Beyond suicide risk, demoralization is associated with a constellation of severe psychological and existential outcomes, including dignity-related distress, hope impairment, psychological well-being deficits, anxiety disorders, mood disorders, and sleep disturbances. People with high demoralization also experience significant impairment in quality of life and work productivity. These outcomes collectively create a downward spiral whereby loss of meaning fuels helplessness and despair, which further compromises the patient’s ability to engage in meaningful activities, maintain social connections, or cope with the demands of illness and treatment [8]. It also adds costs to the medical system in terms of healthcare utilization, exacerbates other forms of physical and psychological pain, and exacts a toll on healthcare providers, who often feel helpless in the face of demoralized patients. Related but distinct is the concept of spiritual distress, defined as loss of meaning, purpose and transcendence, and connection to the moment, to self, to others, to nature, and to the sacred [9]. It is elevated in about a quarter of people with advanced cancer and may also be responsive to PAT [10,11].

### 1.2. Psychedelic Renaissance and Regulation

Psychedelics are a class of psychoactive substances that produce profound changes in perception, mood, and cognition, often inducing altered states of consciousness (see Table 1). Commonly known for their hallucinogenic effects, these substances typically act on the brain’s serotonin receptors, particularly the 5-HT2A receptor, which influence cognition, perception, and emotion [12,13,14,15,16]. Table 1 summarizes common compounds that are often labeled “psychedelics”, recognizing ongoing debate about whether all of these substances should be included in this categorization due to different mechanisms of action and subjective effects [17].

Many of these compounds have been used for millennia in traditional healing and spiritual practices by various cultures [13,15]. In a therapeutic setting, psychedelics are typically administered under controlled conditions, often in combination with psychotherapy, to address mental health conditions such as depression, anxiety, post-traumatic stress disorder (PTSD), and existential distress, particularly in people with terminal illnesses like cancer [18,19].

The “psychedelic renaissance” refers to the renewed scientific interest [20,21] in the therapeutic potential of psychedelics after decades of legal and cultural stigma. Following the prohibition of psychedelics in the 1970s, largely due to sociopolitical pressure and the unethical use of psychedelics in vulnerable populations [22], research in this area was largely dormant. However, beginning in the early 2000s, there has been a resurgence of scientific studies demonstrating the promise of psychedelics in treating various psychiatric conditions, including existential distress in people with advanced cancer [21].

### 1.3. Regulation

In Canada, psychedelics such as psilocybin, LSD, DMT, and MDMA are controlled as Schedule III substances under the Controlled Drugs and Substances Act. This makes these compounds illegal to possess, produce, or distribute without government authorization. Since 2022, healthcare practitioners are authorized to request access to psychedelic drugs through the Special Access Program (SAP), a government program designed to consider requests for access to psychedelics on a case-by-case basis. Approvals are granted in the context of a medical emergency and do not imply that drugs are safe or efficacious. In addition, a class exemption from the Controlled Drugs and Substances Act has also been in place since 2022, allowing some healthcare providers to be exempted from the CDSA under certain circumstances. These exemptions are granted pursuant to the Minister of Health’s authority to do so under Subsection 56(1) of the CDSA, and only in relation to psilocybin and MDMA [23]. For research purposes, anyone conducting clinical trials involving psychedelics must submit a Clinical Trials Application (CTA) to Health Canada, which authorizes conduct of the trial specifying design, safety measures, and intended outcomes. Subsection 56(1) exemptions can also be issued to researchers studying psychedelics in humans or animal models, allowing universities, research institutes, and hospitals to conduct controlled studies involving psilocybin or MDMA under strict supervision. For clinical trials, both a Clinical Trial Application (CTA) and a Subsection 56(1) exemption are required—one to conduct the research and the other to legally possess and handle the compounds.

A particularly poignant issue in the regulatory landscape, often causing moral distress among healthcare providers, is the comparison between the availability of Medical Assistance in Dying (MAID) and restricted access to PAT. While MAID is legally available in Canada for individuals with terminal illness or unbearable suffering, access to therapies like psilocybin, which may relieve existential distress and reduce the demand for MAID, remains limited. Healthcare providers have highlighted the paradox that patients can request life-ending medical procedures but may struggle to access treatments that could reduce their suffering. This comparison has sparked discussions about the ethical implications of Canadian drug policy and the need for expanded access to compassionate care alternatives like PAT [24].

### 1.4. Psychedelic-Assisted Therapy for People with Advanced Cancer

Recent clinical trials have shown the potential for psychedelics, particularly psilocybin, to relieve anxiety, depression, and existential distress in patients with life-threatening advanced cancer. Two notable randomized controlled clinical trials (RCTs) have been conducted in the US, by Griffiths et al. at Johns Hopkins [10], and Ross et al. at New York University (NYU), both publishing primary results in 2016. The Ross et al. [25] study included 31 people with cancer who were randomized in a blinded crossover design to a single high-dose session of psilocybin versus an active control (niacin), administered with psychotherapy. Compared to the niacin group, PAT produced rapid and sustained improvements in depression, demoralization, and hopelessness. Secondary analyses found that PAT was associated with persistent reductions in suicidal ideation and substantial reductions in loss of meaning in life for up to 4.5 years [26].

Griffiths et al. [10] is the largest RCT, to date, and included 51 participants with advanced cancer randomized to either high- or low-dose psilocybin administered in counterbalanced sequence 5 weeks apart, with a 6-month follow-up. Only the high-dose arm resulted in large decreases in clinician- and self-rated measures of depressed mood and anxiety, along with increases in quality of life (QoL), meaning in life, and optimism, and decreases in death anxiety, which were sustained at the 6-month follow-up. Participants attributed these effects squarely to the treatment experience. In both the Johns Hopkins and NYU trials, while participants were described as having “life-threatening” cancers, fewer than half the participants had stage III or IV cancers, emphasizing why additional research focused on populations typically seen in palliative care (with a much higher incidence of advanced diagnoses) is necessary.

A 2021 systematic review and meta-analysis [27] assessing psilocybin-assisted therapy for end-of-life anxiety symptoms included five small studies with a total of 132 participants. Psilocybin was superior to placebo in treating state and trait anxiety at day 1 (Hedges’ g, 0.70, 0.71, respectively) and 2 weeks (1.03, 1.08) after treatment, with large effects. Trait anxiety also improved at 6 months post-treatment (0.84). Similarly, Ross et al. [28] concluded in a review paper that PAT may produce rapid, robust, large, and sustained improvements in cancer-related anxiety, depression, suicidal ideation, existential distress, demoralization, spiritual well-being, and QoL. They also noted psilocybin often led to mystical-type experiences that were considered highly meaningful and spiritual, which partially mediated the anxiolytic and antidepressant effects weeks later, suggesting these mystical experiences may be a potential psychological mechanism of action.

The pace of research in this area continues to gain momentum. In 2023, two open-label pilot studies of PAT for depression in people with advanced cancer were published (with 12 [29] and 30 [30] participants, respectively); both offered group preparation and integration and one 25 mg high-dose psilocybin session. Feasibility was high across the studies, with half of the participants achieving complete remission from depression and most others reporting clinically significant improvements up to 26 weeks post-intervention, with low adverse effects.

Despite promising outcomes, recent psilocybin trials in cancer care remain limited by small sample sizes, homogenous (predominantly White, older, highly educated, higher economic status, US-based) participant cohorts, and short-term follow-up, hindering generalizability to real-world palliative populations [31,32]. Substantial methodological challenges persist, including insufficient diversity, high expectancy, and placebo effects, difficulties maintaining masking due to the unmistakable psychoactive effects of psilocybin, heterogeneous trial designs, lack of data on the safety of combining psychedelics with antineoplastic agents and support medications commonly used in cancer, and inconsistent use or reporting of standardized psychotherapeutic frameworks, all of which confound isolation of drug-specific efficacy and limit scalability for broader oncology practice [32,33,34].

Additionally, despite the rapid pace of research in this area in other parts of the world, no PAT clinical trials have been published in Canada, and stakeholder groups have not been systematically or thoroughly consulted in terms of their concerns and preferences. There is also a lack of diversity in both psychosocial oncology research and psychedelics research generally, including limited inclusion and representation of Black communities, Indigenous peoples, people of color, and people with disabilities [35,36]. One review of 17 psychedelic clinical trials found that 82.5% of all trial participants were White [37]. This lack of diversity also applies to the professionals who designed and implemented the trials. Hence, we are committed to utilizing strategies to mitigate these biases in our study design and implementation by having a leading EDI champion on the research team (Williams), connecting with community leaders from diverse patient groups, and creating welcoming and culturally sensitive materials and therapy elements, with direct input from community members (e.g., [38]).

Given the promising clinical evidence in this area, and the current regulatory and political context in Canada, we argue that it is timely to establish the Canadian Network for Psychedelic-Assisted Cancer Therapy (CAN-PACT; www.canpact.ca (accessed on 1 December 2025)) to address the lack of national infrastructure, clinical trials, and workforce capacity for PAT in cancer care. This initiative will generate clinical evidence, inform policy, enable clinician training, and support equitable access for patients with advanced cancer, directly addressing identified gaps in care and research relevant for real-world implementation in Canada.

## 2. Materials and Methods

### 2.1. Aim

The overall aim of CAN-PACT is to prepare the Canadian oncology healthcare system for the implementation of evidence-based PAT.

### 2.2. Objectives

The objectives of CAN-PACT are the following:

1. Build Capacity: Develop a national PAT research and practice network in cancer care.
2. Research Priorities: Determine key PAT research priorities for people with advanced cancer through stakeholder engagement.
3. Training Programs: Develop and implement PAT training and educational programs/materials for oncology healthcare providers, researchers, patient partners, and policymakers.
4. Pilot Data Collection: Collect pilot and feasibility data to inform design of the larger trial.
5. Clinical Trial: Conduct a multisite RCT of PAT in people with advanced cancer, supported by the Canadian Cancer Trials Group (CCTG).
6. Policy Influence: Inform and influence Canadian healthcare policy to support PAT integration into cancer care.

### 2.3. Procedures

#### 2.3.1. Objective 1: Build the CAN-PACT National Network

To support this objective, we will undertake the following activities:

- Environmental Scan: Conduct a thorough scan of existing research and clinical programs across Canada related to PAT. This systematic assessment will adopt a pan-Canadian scope with targeted regional focus on key regions (British Columbia, Alberta, Ontario, Quebec and the Maritimes) to capture both national trends and regional variations in PAT implementation. The scan will systematically catalog program names, institutional affiliations, focus areas (research versus clinical), funding sources, and ongoing trials or initiatives, while documenting key outputs including publications, clinical trials, treatment protocols, and training materials. By limiting inclusion to credible, active participants in psychedelic research or clinical practices related to cancer, this environmental scan will identify critical gaps in service delivery, opportunities for strategic collaboration, and policy implications that could accelerate PAT integration into mainstream oncology care, where appropriate. While information will be collected on private fee-for-service programs, the emphasis for network membership will be on people working in publicly funded institutions (e.g., hospitals, universities, etc.).
- Network Engagement: Identify and create a directory of potential network members from interested groups including researchers, clinicians, patient advocacy groups, policymakers, and healthcare administrators. Advanced mapping tools (including Miro and Lucid chart) will be employed to visualize network connections and collaborative relationships, enabling strategic identification of influential nodes and potential partnership opportunities within the emerging Canadian PAT ecosystem.
- Network Development: Expand CAN-PACT membership and create collaborative working groups to focus on research, education, clinical care, and policy initiatives. These working groups will be structured around the gaps and opportunities identified through the environmental scan, ensuring an evidence-informed network architecture that maximizes collaborative potential while addressing identified system-level barriers.
- Annual Network Meetings: Hold regular virtual meetings and one large in-person annual meeting to promote collaboration and align goals, facilitating knowledge exchange and coordinated action across the distributed network while maintaining momentum toward shared objectives.

#### 2.3.2. Objective 2: Determine PAT in Cancer Care Research Priorities Through Stakeholder Engagement

**Figure 1:**
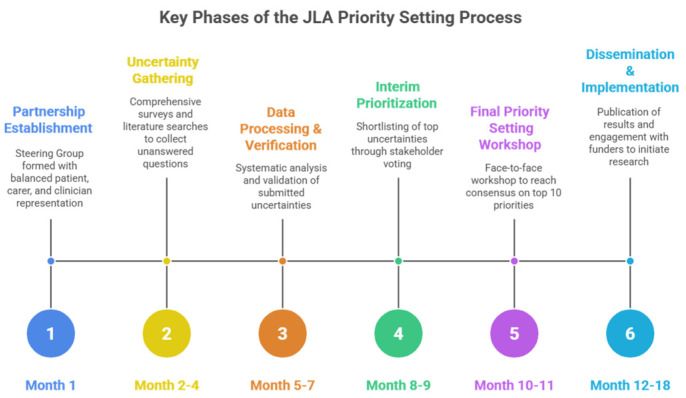
Key phases of the JLA priority setting process.

To achieve this objective, we will conduct a James Lind Alliance Priority Setting Partnership following the established James Lind Alliance (JLA) framework to engage stakeholders in identifying and prioritizing research questions in PAT in cancer care. This process ensures that the priorities of all stakeholder groups are aligned. The JLA is dedicated to ensuring that research addresses issues important to patients, carers, and clinicians. Established in 2004, it facilitates Priority Setting Partnerships (PSPs), where groups collaborate to identify and prioritize key unanswered questions about treatments and care [39]. The JLA method is built on four integral principles: equal involvement of all stakeholder groups, inclusivity in participation, transparency of the process, and commitment to using and contributing to the evidence base. The process brings together patients, carers, and clinicians on equal footing to identify uncertainties in the evidence and jointly prioritize these uncertainties through a structured consensus-building approach. The PSP methodology follows a systematic approach conducted over 12–18 months with several distinct phases (see Figure 1).

- Partnership Establishment: Formation of a Steering Group comprising balanced representation of patients, carers, and clinicians, supported by a JLA Adviser who provides independent facilitation throughout the process.
- Uncertainty Gathering: Collection of unanswered questions through comprehensive surveys targeting patients, carers, and clinicians, supplemented by searches of existing literature for documented uncertainties in guidelines and systematic reviews.
- Data Processing and Verification: Systematic analysis of survey responses to categorize submissions, form indicative research questions, and verify these as true uncertainties by checking against the current evidence base including systematic reviews and clinical guidelines.
- Interim Prioritization: Reduction in the long list of verified uncertainties to a manageable shortlist (typically 25–30 questions) through stakeholder voting, ensuring equal weighting of patient/carer and clinician perspectives.
- Final Priority Setting Workshop: A facilitated face-to-face workshop using an adapted Nominal Group Technique where diverse groups of interested parties engage in structured small group discussions and ranking exercises to reach consensus on the top 10 research priorities.
- Dissemination and Implementation: Publication of results and active engagement with research funders and academic communities to translate priorities into funded research studies.

The process maintains rigor and objectivity through several mechanisms: exclusion of commercial interests and non-clinician researchers from voting, transparent audit trail from original submissions to final priorities, independent facilitation by trained JLA Advisers, and formal verification that all prioritized questions represent genuine evidence uncertainties. The methodology has been continuously refined since its conception and is supported by comprehensive guidance and templates [39]. This established method promotes patient-centered research and helps bridge the gap between research priorities and the practical needs of healthcare providers, patients, and carers, ensuring that the resulting research agenda reflects the shared priorities of those most directly affected by the healthcare challenges being addressed [20].

#### 2.3.3. Objective 3: Develop and Deliver PAT Training and Educational Programs

**Figure 2:**
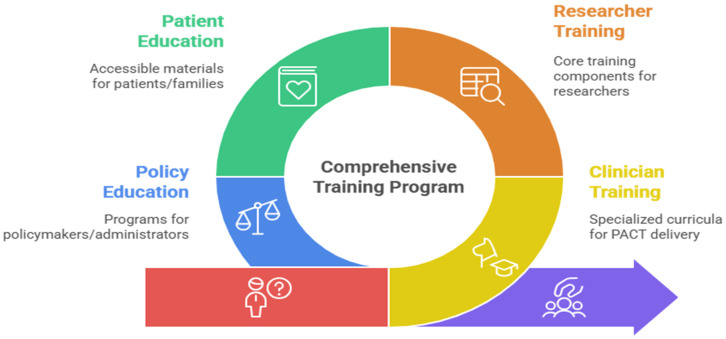
Describing the disparate audiences for PACT education.

To support this objective, we will undertake the following activities for each target audience (described in Figure 2):

- Training for Clinicians, Researchers, and Students: CAN-PACT will build on existing partnerships and resources, with input from our Education subcommittee, to create specialized curricula for PAT in cancer care [40] (e.g., Adapting the University of Ottawa Psychedelics programming), drawing from disparate internationally recognized training standards for PAT and guidance documents [20,41]. The curriculum for both clinical and research trainees will incorporate a comprehensive theoretical foundation spanning several core competency areas, including pharmacological and neurobiological fundamentals, current clinical approaches to demoralization and existential or spiritual distress, consciousness and altered state research, Indigenous healing perspectives, and intercultural considerations specific to Canadian healthcare contexts. In parallel, research trainees (including graduate students and postdoctoral fellows) will receive structured education in the design, conduct, and analysis of psychedelic-assisted trials in oncology. This will include training in protocol development, selection and validation of psychosocial and clinical outcome measures, trial methodology for complex interventions, data management and statistical analysis plans, and integration of mechanistic (e.g., biomarker, digital health) sub-studies within pragmatic trial designs. Trainees will also gain experience in patient-oriented research methods, including stakeholder engagement, priority setting, and co-design of study procedures with people with lived experience, as well as knowledge translation strategies tailored to clinicians, patients, and policymakers.
- Clinical trainees in cancer care designated to deliver the intervention will also receive structured, standardized training in PAT delivery. A formal Mindfulness-Based PAT (MB-PAT) training curriculum is currently in development and will incorporate internationally recognized standards for psychedelic-assisted therapy training combined with elements of mindfulness-based cancer recovery [42] informed by the writings of Shapiro and Carlson [43]. This comprehensive manual will specify the following: (1) detailed protocols for group preparation including specific mindfulness-based training, psychoeducation on PAT mechanisms and treatment expectations, and developing the therapeutic alliance; (2) exact dosing procedure and session structure; (3) detailed integration protocols spanning post-session debriefing, mindfulness techniques, meaning-making, and clinical follow-up; and (4) competency benchmarks for therapist certification. Training will be delivered through multiple modalities including didactic instruction, direct observation of experienced practitioners, supervised role-play, and (where appropriate and permitted), closely supervised experiential components, with formal competency assessments and ongoing supervision to support fidelity. This approach emphasizes experiential training opportunities and comprehensive skill development spanning research design, clinical trial methodology, patient engagement, and knowledge translation. Researchers new to this area and research trainees (graduate students and postdoctoral fellows) will take part in similar core training components to clinicians. Training methods and modalities will include, but not be limited to, the following:
- Case Study Discussions: Regular interdisciplinary case study discussions examining the integration of PAT into Canadian psychosocial oncology and palliative care settings, incorporating structured colloquium methodologies that facilitate peer supervision and collaborative learning among multidisciplinary teams.
- Shadowing Opportunities: Structured observation of clinical trials, patient interactions, and experienced practitioners in palliative care, psychosocial oncology, and community partnerships, with systematic documentation and reflection processes to enhance therapeutic skill development through direct observation and post-session analysis.
- Ethics and Regulatory Training: Comprehensive training on Health Canada regulatory compliance, informed consent processes, and ethical considerations for vulnerable populations in psychedelic therapy contexts, incorporating specialized modules on boundary maintenance, crisis management, and cultural sensitivity within the Canadian healthcare framework.
- Skill Development: Clinical competencies, patient communication, regulatory compliance, mentorship capabilities, patient engagement, hands-on experience, and evidence-to-practice translation—all delivered through individualized learning pathways enhanced by peer supervision groups and ongoing professional development requirements.

This integration maintains academic rigor while clearly establishing the evidence-based foundation of both partnerships and their specific contributions to the training program, ensuring alignment with international best practices for psychedelic-assisted therapy education while addressing the unique needs of Canadian oncology and palliative care settings.

- **Patient and Family Education**: Recognizing the critical importance of informed participation and community acceptance, we will develop culturally sensitive, accessible educational materials that address the unique informational needs of patients, families, and advocacy groups. These resources will provide evidence-based information on PAT’s therapeutic mechanisms, potential benefits for existential distress and demoralization syndrome, comprehensive safety profiles, and realistic outcome expectations. Educational materials will be co-developed with patient partners to ensure relevance and accessibility, incorporating diverse perspectives [44] and addressing concerns specific to cancer populations. We will utilize multiple dissemination formats including, but not limited to, infographics, video testimonials and informational FAQs, webinars, and interactive decision support tools, with materials available in multiple languages (beginning with French and English) to enhance equity and inclusion.
- **Policymaker Education**: To facilitate evidence-informed policy development and healthcare integration, we will engage policymakers to better understand their context, needs, and preferences and create targeted educational programs for policymakers, healthcare administrators, and regulatory bodies, with input from our policy subcommittee. These initiatives will emphasize the growing evidence base for PAT efficacy, as well as limitations, necessary safety protocols and risk mitigation strategies, economic considerations including cost-effectiveness analyses, and implementation frameworks for integration into publicly funded healthcare systems. Educational offerings will include policy briefs synthesizing current research, stakeholder roundtables bringing together researchers and decision-makers, and customized presentations addressing jurisdiction-specific regulatory and implementation challenges.

#### 2.3.4. Objective 4: Pilot Data Collection on Key Outcomes

The CAN-PACT pilot study will be a methodologically sophisticated feasibility assessment designed to evaluate key psychosocial outcomes in advanced cancer patients receiving PAT within a 2 × 2 factorial framework (see Table 2), using the Multiphase Treatment Optimization Strategy (MOST) framework [45]. This pilot investigation will simultaneously assess the independent and combined effects of high-dose psilocybin therapy and intensive mindfulness-based preparation and integration protocols.

Notably, sessions will be offered in a group format, with hybrid in-person/virtual delivery of preparation and integration sessions, and an in-person group-based dosing session. A similar group-based model has successfully been used in two small open-label studies of PAT among people with cancer [29,30].

As a primary objective, the pilot will systematically evaluate operational feasibility metrics, including referral rates, eligibility proportions, enrollment, consent, randomization acceptance, protocol adherence across preparation sessions, dosing experiences, and integration sessions, alongside questionnaire completion rates and loss-to-follow-up patterns over the 6-month study period. These metrics are essential for informing the design of subsequent definitive trials and ensuring the intervention’s translational potential within healthcare systems.

Secondary endpoints from the pilot study will aim to estimate the potential impact of PAT on key psychosocial symptoms in people living with advanced cancer, with a particular focus on demoralization, existential distress, QoL, and death anxiety. Exploratory outcomes include the evaluation of spiritual well-being, meaning-making, and mindfulness capacities. In addition to capturing the severity and clinically meaningful change in these core symptoms, this study will explore experiential factors, such as the occurrence of mystical-type experiences, which may contribute to therapeutic benefit. This multidimensional assessment strategy is designed to clarify both the clinical impact of therapy and the underlying mechanisms that support lasting psychological improvement in this population.

Finally, the pilot study will evaluate a Mindfulness-Based PAT (MB-PAT) intervention as a candidate configuration that integrates mindfulness training within preparation and integration phases, drawing curriculum from the evidence-based Mindfulness-Based Cancer Recovery (MBCR) program [46]. This represents the first empirical evaluation of mindfulness enhancement in PAT for people living with cancer in Canada, addressing a critical gap in the current literature where psychotherapeutic components have received limited systematic investigation. The MB-PAT approach utilizes the IAA mindfulness model (Intention, Attention, Attitude) by Shapiro and Carlson [43] in conjunction with the MBCR curriculum to provide structured mindfulness training that may enhance therapeutic outcomes through shared mechanisms of action with psilocybin, including cognitive flexibility enhancement and openness to experience. Critically, the pilot study will empirically test whether this specific combination produces additive, synergistic, or distinct benefits compared to psilocybin without adapted MBCR.

Notably, however, this pilot study remains in active development and is subject to iterative refinement based on emerging evidence from network environmental scans, evolving academic literature, patient and public involvement, and regulatory guidance updates. The adaptive approach ensures the incorporation of best practices as they emerge while maintaining scientific rigor and patient safety standards. The pilot study will prioritize core psychosocial outcome collection on all participants. Optional exploratory physiological and digital biomarker data collection (e.g., inflammatory cytokines, gut microbiome composition, cortisol, actigraphy, etc.) will be implemented where logistically feasible and will be supported by dedicated funding streams. These mechanistic measures are hypothesis-generating, motivated by emerging evidence linking inflammation, microbiome composition, autonomic regulation, and psychedelic therapy outcomes [47,48,49]. Biomarker collection will be limited to interested sub-samples or specific sites to minimize participant burden and prevent interference with primary psychosocial outcome collection and trial feasibility. Formal decisions regarding which biomarker and physiological assessments to include in the definitive RCT will be made after critical review of pilot data and consultation with patients and clinicians through the priority-setting process and pilot participant feedback, ensuring that exploratory science never jeopardizes robust evaluation of the primary trial objective.

The systematic evaluation of these key primary and secondary outcomes will provide crucial preliminary data on feasibility parameters, effect sizes, and variability estimates necessary for designing the subsequent multi-site Phase III randomized controlled trial, while contributing to the growing evidence base supporting PAT as a promising intervention for cancer-related existential distress.

#### 2.3.5. Objective 5: Conduct a Multisite RCT of PAT for People Living with Cancer

This multi-site trial underwent peer-review through the Canadian Institutes of Health Research (CIHR) in 2022, 2023, and 2024 as an operating grant, where it received partial funding. It was also included as part of the larger Canadian Cancer Society Breakthrough Team Grant, where we received full funding for the trial, as well as the additional work described herein. Given the other components of the Network that will now occur before the multi-site trial, including the research priority setting and pilot trial, the design and methods herein are subject to change.

The original trial submitted to CIHR involved comparing high-dose (25 mg) to low-dose (1 mg) psilocybin administered alongside mindfulness-based preparation and integration therapy in approximately 200 participants with advanced (Stage III or IV) cancer. The trial would assess demoralization syndrome as the primary outcome, with secondary outcomes including spiritual well-being, fear of death and dying, meaning in life, anxiety, depression, QoL, mindfulness capacities, and mystical experiences. Exploratory analyses examining inflammatory and gut microbiome biomarkers and wearable device data were proposed to elucidate potential mechanisms of therapeutic action and identify predictors of treatment response; however, collection would be restricted to sites and sub-samples with dedicated resources to ensure that mechanistic investigations do not compromise the feasibility or quality of core psychosocial outcome data collection across the trial network.

Building upon existing cancer clinical trial infrastructure, this multisite RCT would ideally be nested within the Canadian Cancer Trials Group (CCTG) network. This established framework provides critical operational advantages including standardized randomization procedures, experienced clinical research associates at each site, and comprehensive data safety monitoring through the CCTG Data Safety Monitoring Committee. The infrastructure ensures robust trial conduct across diverse geographical regions while maintaining consistency in protocol implementation and data quality. Running a trial through CCTG requires approval of the study design by the CCTG Clinical Trials Committee. The trial will be submitted to this process once the design is finalized. CCTG-affiliated investigators and patient partners are members of the CAN-PACT network and have been involved with the project since the inception of this work.

Unlike previous PAT trials conducted primarily in academic research settings, this study will incorporate pragmatic effectiveness elements by training publicly funded, hospital-based palliative care and psychosocial clinicians as therapists. We propose that this ‘within-current-clinical-settings’ capacity building approach will address implementation barriers and increase the likelihood of sustainable access to PAT within the Canadian healthcare system following trial completion, should results warrant. In this regard, the trial aims to specifically address diversity and inclusion challenges that have characterized previous psychedelic research, implementing targeted recruitment strategies and culturally sensitive intervention adaptations developed with community and patient partner input.

The final trial design will be substantially informed by results from the priority setting partnership (Objective 2) and the pilot feasibility study (Objective 4), as well as evolving best practices in the field, ensuring optimal protocol refinement based on preliminary efficacy signals, treatment optimization, safety profiles, and operational considerations. Key design parameters subject to modification include sample size, inclusion/exclusion criteria, intervention dosing and session protocols, the type and length of preparation and integration therapy, and outcome measure selection. This adaptive approach maximizes the probability of trial success while ensuring scientific rigor and participant safety throughout the definitive efficacy evaluation. Finally, the integration of novel digital and physiological biomarker collection represents an innovative approach to understanding mechanisms of action and developing personalized treatment algorithms for future clinical implementation.

#### 2.3.6. Objective 6: Influence Canadian Healthcare Policy on PAT

Building upon the previous objectives, CAN-PACT will implement a comprehensive policy engagement strategy based on stated needs and preferences of identified policymakers across relevant provincial/territorial and federal agencies. These may leverage evidence-based policy briefs and a series of workshops tailored to provincial/territorial and federal decision-makers, outlining the evidence supporting PAT, its potential benefits for patients, and its safety and effectiveness for people living with cancer. Relevant policy documents will synthesize emerging research findings from network objectives alongside international regulatory precedents that have advanced psychedelic medicine policies. The workshop series will utilize structured consensus-building methodologies to facilitate meaningful dialogue between researchers, clinicians, patients, and policymakers, ensuring that regulatory frameworks are informed by both scientific evidence and real-world implementation considerations.

The systematic integration of policymakers into the Priority Setting Partnership (PSP) process represents a novel approach to healthcare policy development, ensuring that regulatory frameworks are directly informed by patient and clinician priorities. This methodology provides a robust, evidence-based foundation for policy recommendations by establishing shared priorities across all stakeholder groups, thereby enhancing the legitimacy and acceptance of proposed regulatory changes. The PSP approach has demonstrated value in addressing complex healthcare challenges where multiple perspectives must be reconciled to develop effective policy solutions.

Through direct engagement with federal and provincial/territorial regulatory bodies, leveraging CAN-PACT members’ existing advisory roles with policy groups, the network will contribute to the development of evidence-informed regulatory pathways that balance patient access with safety considerations. This includes advancing discussions around controlled access programs, training and certification requirements for healthcare providers, and quality assurance standards for psychedelic therapies in cancer care settings, paying close attention to PAT context and updates from Health Canada to navigate regulatory challenges in the approval of psilocybin and other psychedelics for therapeutic use in cancer care. The network’s embedded regulatory expertise and established relationships with policymakers position it uniquely to translate research findings into actionable policy recommendations that can facilitate safe, effective, and sustainable implementation of PAT within Canadian oncology healthcare systems.

### 2.4. Patient and Public Involvement

Patient engagement is integral to the CAN-PACT project, grounded in principles of meaningful partnership, shared decision-making, and inclusivity. Individuals with lived experience of cancer—including patients, family members, and caregivers—are actively involved as co-investigators, participating as voting members on the Steering Committee and throughout the project lifecycle. Partners both with and without personal experience of psychedelics are included to ensure a broad range of perspectives. Notably, the CCTG’s 2024 Patient Representative Priorities in Cancer Research report identified psychological and holistic oncology as a top patient priority, further underscoring the alignment of CAN-PACT’s aims with what matters most to Canadian cancer patients and caregivers [50]. Their input informs research priority setting, the co-development of patient-centered materials and resources, recruitment strategies, and study design.

Patient partners are also embedded in the clinical trial process, from the earliest research concept through protocol and consent development, center activation, data collection, and analysis to final reporting and dissemination. They contribute directly to study safety oversight as members of the Data Safety Monitoring Committee, reviewing adverse event reports and ensuring ongoing participant well-being. In addition, patient partners play a key role in interpreting study results, co-authoring presentations and publications, and leading knowledge translation activities to reach cancer patient communities and advocacy networks. Comprehensive support—including financial compensation, direct access to a specific staff member (partner liaison), flexible scheduling, and access to participation resources—is provided to enable full and equitable engagement. This integrated approach ensures that patient perspectives and priorities meaningfully inform all phases of the research, fostering authentic partnership and maximizing the relevance and real-world impact of the project on supportive care for people with advanced cancer.

## 3. Conclusions

In summary, CAN-PACT represents an ambitious, multi-phase initiative to develop the research and training infrastructure, evidence base, and policy context needed to evaluate the safety, effectiveness, and real-world implementation potential of psychedelic-assisted therapy for treating demoralization in people living with advanced cancer. This manuscript has outlined the rationale, structure, and planned components of this programmatic research initiative rather than presenting finalized protocols for individual studies.

Through this coordinated, phased approach—encompassing network development, stakeholder-informed priority setting, training initiatives, feasibility piloting, and rigorous multi-site research—CAN-PACT aims to establish the evidence base and infrastructure necessary for potential implementation of PAT in Canadian cancer centers, should research findings support efficacy, safety, and feasibility in real-world cancer care settings. Additionally, the pathway from research evidence to sustained clinical implementation will require ongoing work in regulatory approval, policy development, workforce training, and systems integration beyond the scope of this research program.

The CAN-PACT initiative is a vital step towards safely integrating evidence-based PAT into Canadian cancer care, driven by patient demand and promising evidence about the potential role of psychedelics in addressing distress experience by people living with advanced cancer. By building a national research and clinical network, determining research priorities, conducting pilot studies, and ultimately running a large multisite trial, this project will generate the evidence needed to determine whether PAT is a viable option for alleviating the psychosocial burden of advanced cancer. We will work with administrators and regulators to align Health Canada, provincial/territorial, and institutional/local policy with research evidence and, if appropriate, ultimately provide accessible and timely access to evidence-based, effective, and safe PAT protocols for people with advanced cancer across Canada.

## Figures and Tables

**Table 1:** Describing well-known classical and atypical psychedelics.

Compound	Common Name/Source	Mechanism of Action	Reported Psychological Effects in Humans	Common Adverse Effects
Psilocybin	“Magic mushrooms” (Psilocybe genus)	Psilocybin is dephosphorylated to psilocin, which activates 5-HT2A receptors on glutamatergic pyramidal neurons in the neocortex, thalamus, and other key brain regions, causing widespread desynchronization of brain networks including the default mode network and anterior hippocampus.	Profound mystical experiences, altered perception of time and space, enhanced introspection and emotional processing, ego dissolution, visual hallucinations (eyes closed), enhanced connectedness to nature and others, spiritual insights, lasting improvements in well-being and life satisfaction. Effects typically last 4–8 h.	Transient anxiety, nausea, headache, elevated blood pressure, and post-session headache. Rarely any persistent long-term medical or psychiatric sequalae.
Lysergic acid diethylamide (LSD-25)	“Acid” Semi-synthetic psychedelic	LSD potently activates 5-HT2A, 5-HT2B, and 5-HT2C serotonin receptors, as well as dopamine D2/D3/D4 and α-adrenergic receptors; its slow dissociation from 5-HT2A receptors prolongs signaling duration and downstream effects.	Intense visual and auditory hallucinations, altered perception of time, synesthesia (blending of senses), heightened creativity, profound introspective insights, ego dissolution, enhanced emotional sensitivity, lasting changes in personality openness, spiritual experiences. Effects typically last 8–12 h.	Can result in anxiety, perceptual changes, nausea, modest heart rate/blood pressure increases, and rarely, longer psychotic or delusional episodes in at-risk populations.
N,N-Dimethyltryptamine (DMT)	Ayahuasca brew component Psychotria Viridis leaves	DMT is a tryptamine that activates 5-HT2A, 5-HT1A, and dopamine D2/D3/D4 receptors; it is not orally bioavailable due to rapid MAO-A metabolism and induces dendritic proliferation in neurons.	Intense but short-duration mystical experiences, ego dissolution, profound spiritual insights, life-changing perspectives on death and meaning. Effects typically last 15–30 min (smoked DMT), peak effect 1–2 h lasting 6–8 h (ayahuasca tea by oral ingestion).	Can lead to rapid-onset anxiety, confusion, intense but short-lived visual effects, with nausea and raised vital signs especially in oral (ayahuasca) use.
5-MeO-DMT	Found naturally in the Bufo alvarius toad	5-MeO-DMT is a non-selective serotonin receptor agonist with highest affinity for 5-HT1A receptors and secondary activity at 5-HT1B, 5-HT1D, 5-HT6, 5-HT7, and 5-HT2C receptors. It readily crosses the blood–brain barrier and distributes widely throughout the brain, producing behavioral effects primarily through 5-HT1A receptor activation while disrupting medial prefrontal cortex oscillations and reducing visual cortex activity.	Users report transcendent experiences involving ego dissolution and non-dual awareness, with emotions ranging from love and awe to panic and terror. Notably, visual effects are often absent; instead, users describe “content-free” states of emptiness or void. When smoked, effects peak within 2–5 min, last 15–20 min, and resolve by 30 min; 90% report positive/transcendent experiences, with 57% meeting criteria for complete mystical experience.	Acute effects include fear, anxiety, paranoia, nausea, vomiting, trembling, and chest/abdominal pressure, with approximately 37% experiencing challenging psychological and somatic effects. Delayed effects (up to 1 week) include muscle tension, sleep difficulties, and flashbacks, while rare cases of psychosis and memory loss have been reported.
Mescaline	“Mescal” Peyote and San Pedro cacti	Mescaline activates 5-HT2A, 5-HT2B, and 5-HT2C serotonin receptors and dopamine D2/D3/D4 receptors while remaining inert at D1/D5 receptors.	Enhanced visual perception with vivid colors and patterns, deep introspection and emotional insights, connection to nature, enhanced empathy, minimal visual distortions compared to other psychedelics. Effects typically last 8–12 h.	Can lead to nausea, anxiety, perceptual alterations, and mild cardiovascular stimulation, with rare persistent psychiatric symptoms.
MDMA	“Ecstasy” (3,4-methylenedioxy-methamphetamine)	MDMA acts primarily through serotonin reuptake inhibition rather than direct serotonin receptor activation, showing minimal 5-HT2A receptor engagement compared to classical psychedelics.	Increased empathy and emotional openness, enhanced social connection and bonding, reduced fear and defensiveness, heightened tactile sensitivity, mild visual enhancements, increased energy and euphoria, enhanced therapeutic communication and trauma processing. Effects typically last 3–6 h.	Can produce jaw clenching, dry mouth, mild hyperthermia, increased heart rate, transient anxiety or paranoia, serotonin syndrome in rare cases, and low mood after use.
Ketamine	“K” Dissociative anesthetic	Ketamine is a dissociative that engages glutamate-dependent homeostatic plasticity mechanisms and modulates limbic connectivity, producing network desynchronization patterns similar to classical psychedelics.	Dissociative effects creating a sense of detachment from body/environment, rapid antidepressant effects, altered perception of reality, out-of-body experiences, reduced rumination and negative thought patterns, pain relief, dream-like states. Effects typically last 1–2 h.	Induces dissociation, dizziness, increased blood pressure, and, at higher or repeated doses, can cause agitation, confusion, or urinary issues. Substantial addiction and abuse liability.
Ibogaine	Found in the root bark of Tabernanthe iboga (‘Iboga’)	Ibogaine acts on multiple targets—including NMDA, sigma-2, opioid, and serotonin systems, as well as nicotinic receptors—to reduce drug-seeking, but increases cardiotoxicity risk via hERG channel blockade and QT prolongation.	Ibogaine produces a multi-phase subjective experience beginning with an intense, dream-like state featuring vivid imagery and deep introspection, followed by a reflective, emotionally neutral period, and ending with a residual phase of heightened awareness and possible sleep disruption. Effects can last between 24 and 72 h.	Can cause nausea, tremors, ataxia, cardiac risks (QT prolongation and arrhythmias), rare mania or seizures, and in rare cases, has resulted in fatal outcomes.

**Table 2:** Describing 2 × 2 factorial framework.

		Mindfulness	
		Intensive	Minimal
Psilocybin	High dose	Combines masked high-dose psilocybin with intensive mindfulness-based preparation and integration, potentially maximizing therapeutic effects.	Explores the impact of masked high-dose psilocybin with minimal preparation, isolating the potential effect of psilocybin.
	Low dose	Combines masked low-dose psilocybin with intensive mindfulness-based preparation and integration, isolating the effect of mindfulness.	Investigates baseline effects of masked low-dose psilocybin with minimal preparation, serving as a control/placebo comparator.

## Abbreviations

The following abbreviations are used in this manuscript:

CAN-PACT	CAnadian Network for Psychedelic-Assisted Cancer Therapy
CCTG	Canadian Cancer Trials Group
CDSA	Controlled Drugs and Substances Act
CIHR	Canadian Institutes of Health Research
CTA	Clinical Trials Application
DS	Demoralization Syndrome
DMT	N,N-Dimethyltryptamine
EDI	Equity, Diversity, and Inclusion
IAA	Intention, Attention, Attitude (mindfulness model)
JLA	James Lind Alliance
LSD	Lysergic acid diethylamide
MAID	Medical Assistance in Dying
MB-PAT	Mindfulness-Based PAT
MDMA	3,4-methylenedioxy-methamphetamine
NYU	New York University
PAT	Psychedelic-Assisted Therapy
PSP	Priority Setting Partnership
PTSD	Post-Traumatic Stress Disorder
QoL	Quality of Life
RCT	Randomized Controlled Trial
SAP	Special Access Program
